# Vertical Transmission of *Pneumocystis jirovecii* in Humans 

**DOI:** 10.3201/eid1501.080242

**Published:** 2009-01

**Authors:** Marco A. Montes-Cano, Magali Chabe, Maria Fontillon-Alberdi, Carmen de la Horra, Nieves Respaldiza, Francisco J. Medrano, Jose M. Varela, Eduardo Dei-Cas, Enrique J. Calderon

**Affiliations:** Virgen del Rocío University Hospital, Seville, Spain (M.A. Montes-Cano, M. Fontillon-Alberdi, C. de la Horra, N. Respaldiza, F.J. Medrano, J.M. Varela, E.J. Calderon); Center for Biomedical Research in Epidemiology and Public Health, Seville (M.A. Montes-Cano, M. Fontillon-Alberdi, C. de la Horra, N. Respaldiza, F.J. Medrano, J.M. Varela, E.J. Calderon); Lille Pasteur Institute, Lille, France (M.A. Montes-Cano, M. Chabe, E. Dei-Cas)

**Keywords:** Pneumocystis jirovecii, vertical transmission, PCR, letter

**To the Editor:** Currently, animal and human studies favor an airborne transmission pattern for *Pneumocystis* pneumonia (*1*). However, the early age of acquisition of *Pneumocystis* spp. in different mammals, including humans, warrants study of vertical/transplacental transmission as an additional route of transmission of this stenoxenic microorganism.

Available studies on transplacental transmission of *Pneumocystis* spp. suggest that it varies among mammal species on the basis of the type of placenta (*2*). Transplacental transmission of *Pneumocystis* spp. has been demonstrated in rabbits (*2*,*3*), but it seems not to occur in rats and mice that have severe combined immunodeficiency (*1*). In humans, transplacental transmission was first suggested by a few reports of *Pneumocystis* pneumonia in neonates published before the AIDS epidemic (*4*). Recently, a controversial case of vertical transmission of *P*. *jirovecii* was reported: an infection in the lungs of a fetus of an HIV-positive mother with *Pneumocystis* pneumonia (*5*). However, the study did not identify the organisms as *Pneumocystis* spp., and a subsequent fluorescein-labeled monoclonal antibody test yielded negative results (*6*).

The present study was conducted to evaluate transplacental transmission of *P*. *jirovecii* by molecular techniques. Placentas and lung tissues of aborted fetuses from immunocompetent women who had miscarriages were studied. To enhance specificity of the study, we used 2 genetic loci in *Pneumocystis* spp. DNA: the mitochondrial large subunit rRNA (mtLSU-rRNA) gene and the gene encoding for dihydropteroate synthase (DHPS). We analyzed 40 paraffin-embedded tissue blocks from the placentas and lungs of 20 fetuses at 28 ± 8 weeks of gestation. The study was reviewed and approved by the ethical committee of University Hospital, Seville, Spain.

DNA was extracted from a mixture of five 10-μm sections of each tissue block. Histologic sections were processed by using xylene and ethanol for paraffin removal and were then rehydrated. DNA was extracted by using the QIAamp DNA Mini Kit (QIAGEN, Hilden, Germany) following the manufacturer’s instructions.

DNA amplification at the mtLSU-rRNA locus was conducted by using nested PCR as described (*7*). Samples identified as positive by this PCR were amplified by using primers DHPS-3 and DHPS-4 to detect the DHPS gene (*7*). To prevent false-positive results caused by contamination, pipettes with filters were used at all stages. DNA extraction, preparation of the reaction mixture, PCR amplification, and detection were conducted in different areas under a laminar flow hood. Positive and negative controls were included in each reaction. All experiments were repeated at least twice.

*P*. *jirovecii* genotypes can be characterized by identifying polymorphisms at the mtLSU-rRNA locus (positions 85 and 248) and at the DHPS locus (positions 55 and 57). Amplicons from all samples that yielded positive PCR results for the 2 loci were sequenced directly from both ends by using a model ABI 377 automated sequencer and an ABI prism Dye Terminator cycle sequencing kit (Applied Biosystems, Foster City, CA, USA) following the manufacturer’s instructions. The derived mtLSU-rRNA and DHPS gene sequences were compared with sequences available in databases by using the *National Center for Biotechnology Information* (Bethesda, MD, USA) BLAST program (http://blast.ncbi.nlm.nih.gov/Blast.cgi).

The mtLSU-rRNA fragment was amplified from 11 lung and 8 placenta samples. Simultaneous DNA amplification of 2 loci of *P*. *jirovecii* was observed in lung tissue samples from 7 (35%) of 20 fetuses and from 1 (5%) of 20 placenta samples. Sequencing of the mtLSU-rRNA gene showed 3 polymorphisms, and DHPS gene analysis showed only wild-type genotype in all samples (Table).

**Table:** *Pneumocystis jirovecii* genotypes in samples identified as positive by nested PCR for 2 loci from fetal lung tissues and placenta samples*

Case	Lung tissues			Placenta tissues	
	mtLSU-rRNA genotype	DHPS genotype		mtLSU-rRNA genotype	DHPS genotype
C1	3	1		–	–
C2	1	1		1	–
C3	3	1		–	–
C4	3	1		–	–
C5	2	1		1	–
C6	1	1		3	–
C7	1	1		–	–
C8	–	–		1	1
C9	3	–		–	–
C10	–	–		3	–
C11	3	–		1 and 3	–
C12	1 and 3	–		1	–
C13	–	–		1	–
C14	3	–		–	–
C15-C20	–	–		–	–

*Genotype 1, polymorphism 85C/248C in mitochondrial large subunit (mtLSU)–rRNA gene and 55 Trh/57 Pro indihydropteroate synthase (DHPS) gene; genotype 2, polymorphism in 85A/248C; genotype 3, polymorphism in 85T/248C.

Our results provide molecular evidence of *P*. *jirovecii* transplacental transmission in humans. No available data on the development of *Pneumocystis* organisms in female genital organs was provided (*8*). In contrast, morphologic and molecular evidence of hematogenous dissemination of *P*. *jirovecii* from infected lungs has been provided by many authors (*8*). *Pneumocystis* DNA has been documented in blood or amniotic fluid samples from pregnant rabbit does (*3*), in which transplacental transmission of *Pneumocystis* spp. occurred. In humans, *P*. *jirovecii* colonization was observed in 5 (15.5%) of 33 pregnant women in their third trimester (*9*). These data suggest that physiologic immunodepression associated with pregnancy may favor *Pneumocystis* spp. colonization and mother-to-fetus transmission of the fungus by the hematogenous route. The transplacental route could enhance transmission of *P*. *jirovecii* independent of environmental hazards.

Isolation of pathogens from an aborted fetus does not necessarily mean that they have caused the death of the fetus because many agents appear to pass through the fetal-placental unit and cause little damage. However, fungal infection is a major worldwide cause of abortion in cattle (*10*), and the surprising high prevalence of *P*. *jirovecii* infection found in dead fetuses in our study emphasizes the need to study the possible role of this fungal organism in human abortion.

Our findings could be of potential clinical importance and could open a new field of research, which should be explored. Further research should assess the scope of the problem and design rational preventive strategies, if necessary.
